# Increased expression of EphA7 correlates with adverse outcome in primary and recurrent glioblastoma multiforme patients

**DOI:** 10.1186/1471-2407-8-79

**Published:** 2008-03-25

**Authors:** Lin-Fang Wang, Emmanouil Fokas, Janko Juricko, An You, Frank Rose, Axel Pagenstecher, Rita Engenhart-Cabillic, Han-Xiang An

**Affiliations:** 1 Department of Radiotherapy and Radiation Oncology, Philipps-University Marburg, Baldingerstr. D-35043 Marburg, Germany; 2 Department of Neuropathology, Philipps-University Marburg, Baldingerstr. D-35043 Marburg, Germany; 3 Department of Emergency Surgery, Union Hospital, Tongji Medical College, Huazhong University of Science and Technology, 430030 Wuhan, People's Republic of China

## Abstract

**Background:**

Malignant gliomas are lethal cancers, highly dependent on angiogenesis and treatment options and prognosis still remain poor for patients with recurrent glioblastoma multiforme (GBM). Ephs and ephrins have many well-defined functions during embryonic development of central nervous system such as axon mapping, neural crest cell migration, hindbrain segmentation and synapse formation as well as physiological and abnormal angiogenesis. Accumulating evidence indicates that Eph and ephrins are frequently overexpressed in different tumor types including GBM. However, their role in tumorigenesis remains controversial, as both tumor growth promoter and suppressor potential have been ascribed to Eph and ephrins while the function of EphA7 in GBM pathogenesis remains largely unknown.

**Methods:**

In this study, we investigated the immunohistochemical expression of EphA7 in a series of 32 primary and recurrent GBM and correlated it with clinical pathological parameters and patient outcome. In addition, intratumor microvascular density (MVD) was quantified by immunostaining for endothelial cell marker von Willebrand factor (vWF).

**Results:**

Overexpression of EphA7 protein was predictive of the adverse outcome in GBM patients, independent of MVD expression (p = 0.02). Moreover, high density of MVD as well as higher EphA7 expression predicted the disease outcome more accurately than EphA7 variable alone (p = 0.01). There was no correlation between MVD and overall survival or recurrence-free survival (p > 0.05). However, a statistically significant correlation between lower MVD and tumor recurrence was observed (p = 0.003).

**Conclusion:**

The immunohistochemical assessment of tissue EphA7 provides important prognostic information in GBM and would justify its use as surrogate marker to screen patients for tyrosine kinase inhibitor therapy.

## Background

The incidence of brain tumors worldwide is about 7 in 100,000 per year [1,2]. Glioblastoma multiforme (GBM), the most aggressive tumor among malignant gliomas, is the most common primary brain tumor in adults and represents a significant source of cancer-related death. GBM usually recurs despite the most aggressive treatment via surgical resection of the tumor followed by radiation and/or chemotherapy [1,2]. The poor prognosis of patients with GBM (median survival ranging from 9 to 12 months, 5-year survival rate close to 0%) mandates the exploration of novel molecular mechanisms that might contribute to the pathogenesis of this disease and its resistance to therapy with the purpose of therapeutic targeting [1-3].

Receptor tyrosine kinases (RTKs) are known to be important regulators of cellular growth controlling cell proliferation, differentiation and migration [4,5]. The Eph receptors and their ligands, ephrins, represent the largest known family of RTKs. Their role has been largely studied during the development of nervous system. They are involved in the development of central nervous system, including axon guidance, axon fasciculation, neural crest cell migration, hindbrain segmentation, vasculogenesis and neuronal cell survival during embryonic development [6-13]. Eph receptors and ephrin ligands are classified into A and B subfamily, on the basis of their sequence, homologies, structures, and binding affinities. EphA receptors bind the glycosylphosphatidylinositol (GPI)-anchored ephrin-A ligands, whereas EphB receptors bind the transmembrane ephrin-B ligands, whose cytoplasmic domain is capable to engage in various signaling activities; an exception is the EphA4 receptor that binds ephrin-B2 and ephrin-B3 as well as ephrin-A ligands [14-16]. Moreover, these RTKs have the ability to induce both forward and reverse (bi-directional) signaling between adjacent interacting cells.

To date, various studies have investigated the involvement of the Eph-RTKs in several pathogenetic processes in the nervous system. EphB2 and ephrin-B2 signaling participate in the glial scarring process after spinal cord trauma [17]. The phosphorylation ratio of R-Ras was closely linked to the phosphorylation ratio of EphB2 in glioblastoma tissues [18]. Additionally, the phosphorylation ratio of EphB2 is an important mechanism that mediates glioma cell migration and invasion [19]. Ephrin-B2 and EphB4 were overexpressed by endothelial cells of human malignant gliomas [20]. Ephrin-B3 was also demonstrated as an important factor regulating glioma cell invasion through Rac1 GTPase [21]. EphA2 protein was overexpressed in GBM and anaplastic astrocytoma tissues and was identified as a novel target for the development of glioma vaccines [22,23]. Another group confirmed overexpression of EphA2 expression in GBM cells, probably through decreased interaction between EphA2 receptor and its inhibitory ligand ephrin-A1 in malignant cells [19].

EphA7 (formerly known as Mdk1/Ebk/Ehk) is highly conserved in vertebrates from fish to human [24]. It is widely expressed in embryonic tissues, especially developing central nervous system [25]. EphA7 cooperates with other EphA receptors in cell signaling, but in contrast to other Eph receptors, it contains two developmentally regulated isoforms: a full-length version containing the intracellular tyrosine kinase domain and a truncated form that lacks this domain [26]. Immunoreactivity for the full-length wild type receptor is found in all cell populations expressing EphA7 mRNA in mouse embryo heads and developing brain, while the truncated EphA7 is absent in the embryos. Interestingly, both isoforms show striking distributions in adult mouse brain [27]. The full-length EphA7 is strongly expressed in neuropil; in contrast the truncated EphA7 is conspicuous on cell bodies and proximal dendrites of a limited number of neuronal types [28]. The truncated form of EphA7 acts as a dominant negative antagonist, suppressing tyrosine phosphorylation of the full-length EphA7 receptor and shifts the cellular response from repulsion to adhesion. Additionally, EphA7 is probably essential during closure of the neural folds, since EphA7-null mice displayed lack of the neural folds resembling anencephaly in man [29]. Moreover, EphA7 has been identified as an important molecular cue expressed after spinal cord injury, implicated in glial apoptosis [30]. Recent work indicated EphA7 as an important mediator of neural progenitor apoptosis during brain development [31]. However, little is currently known about its role in brain tumor angiogenesis and pathogenesis.

In the present study, we investigated the immunohistochemical expression of EphA7 and correlated it with clinical pathological parameters and tumor vascularity. We provide evidence that EphA7 is overexpressed in GBM and suggest that this receptor might be used as a new diagnostic and prognostic marker for further Eph/ephrin targeted molecular cancer therapy.

## Methods

### Tissues

Tumor samples of 32 patients with histologically confirmed GBM, WHO IV, (26 primary GBM, 6 recurrent GBMs and 10 normal brain samples) were obtained from the Department of Neuropathology, Marburg University Hospital, Germany. Approval for immunohistochemical study conduct in this GBM tissue bank had been obtained by the university authorities together with the signed consent of the patients. The patients underwent surgery and received adjuvant radiation therapy combined with chemotherapy using the schema ACNU and VM-26. The first follow-up occurred 6 weeks after therapy was completed. Subsequent follow-ups were scheduled every 3 months. In addition to clinical investigations and monitoring of indices of recurrence, a radiological examination was performed to detect possible relapses. Disease progression was defined according to WHO criteria by either an increase of at least 25% in tumor size or any new tumor identified by CT or MRI scan. Normal brain samples, which included cortex and white matter, were obtained from autopsy cases without any evidences of brain tumor or other brain disease. Totally, five cases of male and five cases of female (age ranging from 27 to 70 years, average: 48.6 years) were obtained.

### Immunohistochemistry

Immunohistochemical studies were performed on formalin fixed, paraffin-embedded tissue. Samples slides were passed through a sequence of Roti-histol (Carl Roth, Karlsruhe, Germany) and graded alcohol and then rinsed in phosphate-buffered saline (PBS). After rinsing with PBS, the slides were treated with 3% hydrogen peroxide in PBS for 15 min at room temperature in order to abolish endogenous peroxidase activity. Subsequently, the slides were treated with 5% blocking serum for 1 hour. Following this, slides were incubated overnight at 4°C with a rabbit anti-human EphA7 polyclonal antibody (H-55) against amino acids of human EphA7 (1:100 dilution; Santa Cruz Biotechonology, Heidelberg, Germany), or rabbit anti-human Factor VIII (von Willebrand Factor, vWF) polyclonal antibody (1:400 dilution; Dako Cytomation, Carpinteria, CA). In negative controls, the primary antibody was replaced with 1 × PBS. The signal was enhanced by using biotinylated polyclonal goat-anti-rabbit IgG with streptavidin-HRP (Dako Cytomation, Carpinteria, CA) for 30 minutes. The colour was developed after 5 minutes incubation with 3,3-diaminobenzidine (DAB) solution and sections were weakly counterstained with hematoxylin for 10 seconds.

### Evaluation of EphA7 expression

The membranous and cytoplasmic expression of EphA7 on tumor cells was assessed at a ×400 magnification. The assigned score first reflects the staining intensity A (0, negative; 1, weak; 2, moderate; 3, high) and second the percentage of positive cells B (0, no positive cells; 1, <25% positive cells; 2, 25 to 50% positive cells; 3, >50% positive cells). An overall score of 3 is defined as positive staining. The scoring was performed separately by two independent observers who were blinded to the clinical data. Any discrepancies were resolved on the conference microscope.

### Evaluation of MVD at "hot spot" of tumor angiogenesis

Tumor angiogenesis can be reflected by MVD in the most vascularised areas of tumor tissue. MVD, as highlighted by factor VIII-related antigen immunostaining, was assessed without knowledge of the patient's clinical outcome, as described by Weidner et al [32]. Briefly, each slide was scanned at low magnification (×100) to identify four areas with the highest density of microvessel (hot-spots). Each hot-spot was then evaluated at high power magnification (×200) for the number of stained microvessels per field in a 0.7386 mm^2 ^surface area. vWF-positive stained blood vessels with a complete lumen as well as cell clusters without lumina were considered as individual microvessels. The final microvessel score was the average of vessel counts from four fields assessed by a high power magnification field (×200).

### Statistical analysis

Survival curves were estimated using the Kaplan-Meier method. The distributions of survival were compared using the log rank test. The chi-square test was employed to determine the association between EphA7 expression intensity on tumor cells and MVD. A p-value < 0.05 was considered to be statistically significant. All statistical analysis was performed using SPSS software.

## Results

### Demographic factors

32 patients with histologically confirmed primary and recurrent GBM, WHO grade IV, were studied. The mean age at diagnosis was 54.3 years (range 31–71). No significant difference in age distribution between male (21 cases) and female (11 cases) was detected. All of the 32 patients showed a relapse between 1 and 22 months after surgery and subsequently died of the disease (median survival 15 months).

### EphA7 immunoreactivity in GBM and normal brain tissues

**Figure 1:**
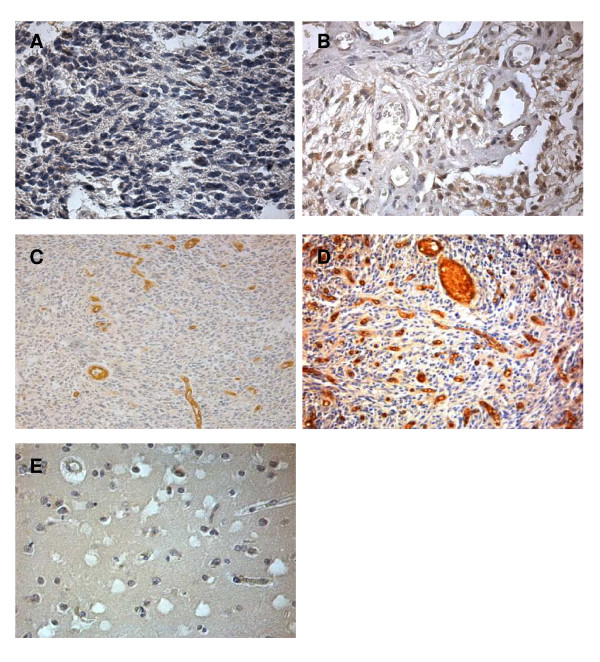
**Immunohistochemical demonstration of EphA7 protein expression and blood vessels in GBM**. Representative examples of GBM showing negative staining in tumor cells (A) or strong membranous and cytoplasmic staining in both tumor cells and endothelial cells (B). MVD in GBM by immunohistochemical staining for vWF, microvessels are represented by brown clusters, which stand out sharply from other tissues. Low tumor vascularity (C) in GBM with low expression of EphA7 as shown A. In contrast, microvessel density was relatively high (D) in GBM with high expression of EphA7 as shown B. Negative staining in normal brain tissue (E). Original magnification, ×400 (A, B, E) and ×200 (C, D).

In 22 of 32 patients, EphA7 immunoreactivity was observed on the prominent membrane and cytoplasm of tumor cells showing different intensities of EphA7 protein. Representative photomicrographs illustrating specimens with negative and strong EphA7 expression in tumor cells are presented in Fig. 1A and 1B, respectively. EphA7 protein expression in glioma cells of 10 normal brain tissues analyzed was undetectable as it was shown in Fig. 1E. Of the 32 GBM analyzed, strong expression of EphA7 (staining intensities of from 6 to 9) was observed in 14 cases (43.7%) of GBM. The staining was specific in both tumor and endothelial cells, with minimal staining of surrounding connective tissues.

### EphA7 associated with MVD in GBM

All tumors were stained with vWF and microvessels were counted as a measure of tumor's angiogenic activity. The median MVD of 30 vessels was treated as a cutpoint. High MVD was noted in areas where overexpression of EphA7 was marked (Table 1). Representative examples of low or high MVD were showed in Fig 1C and 1D, respectively. There was a statistically significant correlation between expression of EphA7 and MVD in the tumors (P = 0.004, Table 1).

**Table 1:** Relationship between EphA7 expression and intratumor microvessel density in 32 primary and recurrent GBM.

	**EphA7 expression**		**P value**
	negative	positive	
MVD			
Low	10	10	
High	0	12	0.004

MVD: microvessel density, N: number of patients. The median MVD of 30 vessels was used as a cutpoint.

### EphA7 immunoreactivity predicted overall survival but not recurrence-free survival

**Figure 2:**
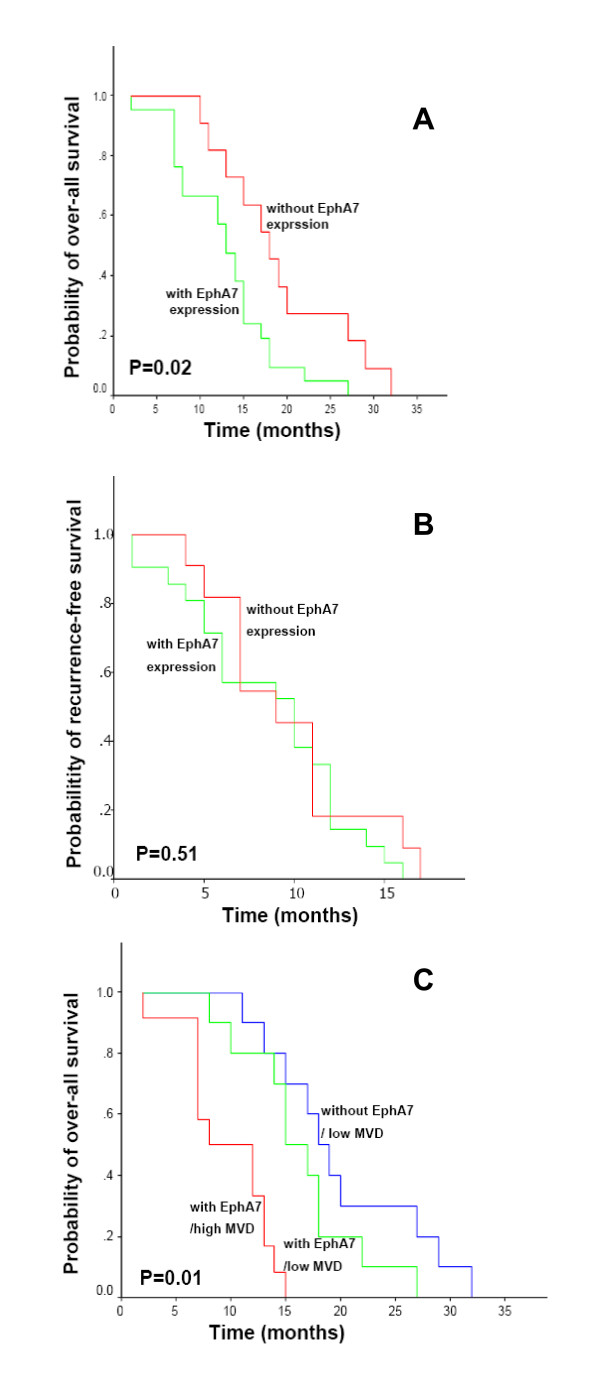
**Kaplan-Meier curve for overall survival and recurrence-free survival in 32 patients based on EphA7 expression and MVD index**. (A) Increased EphA7 expression was significantly associated with dead of disease (*P *= 0.02 by log-rank test), when positive EphA7 (score = 4–9) expressing tumors were plotted against negative EphA7 expressing tumors (score = 0–3). (B) EphA7 expression revealed no significance for recurrence-free survival. (C) EphA7 expression in combination with high MVD showed an inverse outcome (p = 0.01).

The median survival of patients with positive EphA7 expression was reduced in comparison with patients with negative EphA7 expression. EphA7 protein expression showed an inverse correlation with the overall survival (p = 0.02, Fig. 2A). However, the level of EphA7 expression did not emerge as a prognostic factor for recurrence-free survival of GBM patients (p = 0.51, Fig. 2B). There was no correlation between MVD and overall survival or recurrence-free survival (p > 0.05, data not shown). We further explored the prognostic relationship using EphA7 in combination with MVD. The study cohort could be divided into 3 groups based on expression for EphA7 and MVD combination: EphA7(+)/high MVD (n = 12), EphA7(+)/low MVD (n = 10), EphA7(-)/high MVD (n = 0), EphA7(-)/low MVD (n = 10). As shown in Fig. 2C, high density of MVD as well as EphA7 expression predicted for the disease outcome more accurately than Eph variable alone (p = 0.01).

### Clinical features associated with EphA7 or MVD

Statistical correlation was detected between the expression levels of EphA7 or MVD and clinical pathological parameters such as age, gender and tumor status (Table 2). A statistically significant correlation between higher MVD and tumor recurrence was observed (p = 0.003). In addition, positive EphA7 expression was associated with increased age of patients (>55 years, p = 0.003).

**Table 2:** Relationship between EphA7 expression or microvessel count and clinicopathological features of 32 patients with GBM.

	**EphA7 expression**		**P value**	**MVD**		**P value**
Age	N	positive (%)		N	high MVD N (%)	
<= 50	10	3 (30)		11	6 (54.55)	
>50	22	19 (86.36)	0.003	21	16 (76.19)	ns
Gender						
male	21	15 (71.43)		21	15 (71.43)	
female	11	7 (63.64)	ns	11	8 (72.72)	ns
Tumor status						
Primary	26	20 (76.92)		26	22 (84.62)	
Recurrence	6	2 (33.33)	ns	6	1 (16.67)	0.003

MVD: microvessel density, N: number of patients, ns: not significant. The median MVD of 30 vessels was used as a cutpoint.

## Discussion

There is currently an urgent need for development of alternative, effective diagnostic and therapeutic approaches to GBM. The survival of patients with GBM may depend on the identification of novel targets. EphA2 receptor has already been recognized as a potential molecular marker and target in GBM for the development of novel biological therapeutic agents [22,23,33]. Whereas several studies in recent years have clearly indicated that altered expression of Eph receptors and ephrin ligands is associated with increased potential for tumor growth, angiogenesis, metastasis and adverse outcome [34-42], few studies have addressed the role of EphA7 in tumor pathogenicity. By employing immunohistochemical techniques we have found that EphA7 protein is predictive for the outcome of patients with GBM, independent of MVD expression. The data in the present study revealed for the first time a strong correlation between EphA7 overexpression and patient survival.

Hafner C, et al. found that EphA7 is highly expressed in kidney vasculature [43]. The mRNA of EphA7 is strongly upregulated in hepatocellular carcinoma as compared with healthy liver tissue and is downregulated in colon carcinomas. EphA7 is also transcriptionally activated in lung cancer [44]. Furthermore, overexpression of EphA7 protein is frequently found in younger patients and in patients with advanced gastric carcinoma [45]. EphA7 expression is frequently silenced in human colorectal carcinoma by aberrant promoter methylation [46]. EphA7 is located on 6q16.1, a region in close proximity to the chromosome 6 breakpoint found in various types of cancer [47]. Although our findings are not consistent with Wang et al, who found a significant downregulation of EphA7 in colorectal carcinoma [46], they are in line with previous reports reporting a tumor promoter role in lung cancer and hepatocellular carcinoma [43,44], implicating tumor type-specific function for different Eph family members. Eph receptors expressed in different cell types may have opposite effects due to cell-type specific intracellular signaling pathway [4]. Indeed, EphB4 receptor has been identified as a tumor suppressor in breast cancer, through activation of Abl-Crk antioncogenic pathway [48], while the same receptor presented a tumorigenic potential in mesothelioma, favoring uncontrolled cell growth, migration, and tumor progression [49]. Moreover, membrane-bound ephrins trigger Eph receptor phosphorylation, while soluble forms can bind to Eph receptor, but do not trigger receptor activation [50]. Murine and human peripheral lymphocytes secrete a truncated form of EphA7 [51]. Truncated Eph receptors retaining their ligand-binding capacity have been shown to block activation of the full-length receptor [52]. Promoter hypermethylation and silencing of EphA7 in mature B-cell lymphomas may serve to eliminate the inhibitory activity of secreted EphA7 on tumor-promoting EphA7 receptor signaling, thus enhancing tumor cell spread and recruitment of accessory cells able to promote tumor growth [51]. A recent study on signaling pathways involved in EphA7 RTK reported that direct EphA7 knockdown can result in attenuation of ERK1/2 phosphorylation and induce apoptosis of leukemia cells, suggesting the impact of EphA7 on the growth of tumor cells [45]. It is of interest that positive EphA7 expression was closely associated with increased age of patients (>55 years). Whether this is a random finding or not deserves further investigation.

The unfavorable prognostic influence of EphA7 in GBM could be attributed to the well-recognised role of Eph RTKs in tumor angiogenesis. Indeed, in this study a statistically significant correlation between expression of EphA7 and MVD was noted in GBM specimens. Another important observation was EphA7 overexpression in both vasculature as well as tumor cells. The process of angiogenesis plays a central role in tumor growth and in the development of distant metastases by facilitating the entry of cells into circulation [52-55]. A vast biochemical and genetic evidence has implicated the critical role of Eph/ephrin signaling in angiogenesis, despite of VEGFR2 and Tie2 receptors long been recognized as key players in this process [53,54]. Angiogenetic activity can be measured histologically by MVD, which has been shown to be an independent prognostic parameter in various malignancies including gliomas [56-58]. However, other studies on angiogenesis of glioblastomas suggested the limited usage of MVD as prognostic parameter due to the complexity of the microvascular network in GBM [59,60]. Although no correlation between MVD and overall survival or recurrence-free survival was found in our study, we observed a statistically significant correlation between lower MVD and tumor recurrence. Further prospective studies with large numbers of patients are, however, needed to fully clarify the clinical implications of MVD in GBM recurrence.

## Conclusion

Taken together, our data illustrated that EphA7 could be a potential candidate as a prognostic tumor marker and a new targeted therapeutic assessment in primary and recurrent GBM. Based on our findings, there might be a possible relationship between EphA7 and tumor neovascularization. Recent data demonstrating inhibition of angiogenesis through EphA receptor blockade in two different animal tumor models are consistent with our observation [52]. Additional experimental work is necessary to unveil the biologic pathway linking Eph/ephrins with tumor growth in cancer cells and tumor-associated vessels of GBM and further studies are needed before EphA7 becomes established as an important prognostic and predictive tool in GBM. Ultimately, specific EphA7 inhibitors may prove to be of therapeutic value.

## Abbreviations

GBM, glioblastoma multiforme; RTKs, Receptor tyrosine kinases; MVD, microvascular density; vWF, von Willebrand factor.

## Competing interests

The author(s) declare that they have no competing interests.

## Authors' contributions

LFW, EF and JJ carried out the Immunohistochemical studies. AY and FR participated in the design of the study and performed the statistical analysis. AP and HXA participated the evaluation of analysed parameters and tumor pathological characteristics. REC conceived of the study and participated in the design and coordination as well as helped to draft the manuscript. All authors read and approved the final manuscript.

## Pre-publication history

The pre-publication history for this paper can be accessed here:


